# The activation of nicotinic acetylcholine receptors enhances the inhibitory synaptic transmission in the deep dorsal horn neurons of the adult rat spinal cord

**DOI:** 10.1186/1744-8069-3-26

**Published:** 2007-09-25

**Authors:** Daisuke Takeda, Terumasa Nakatsuka, Jianguo G Gu, Munehito Yoshida

**Affiliations:** 1 Department of Orthopaedic Surgery, Wakayama Medical University, 811-1 Kimiidera, Wakayama 641-8510, Japan; 2 Department of Physiology, Kansai University of Health Sciences, Osaka 590-0482, Japan; 3 Department of Physiology, Faculty of Medicine, Saga University, Saga 849-8501, Japan; 4 Brain Institute and Department of Oral Surgery, Division of Neuroscience, College of Dentistry, University of Florida, Gainesville, Florida 32610, USA

## Abstract

Somatosensory information can be modulated by nicotinic acetylcholine receptors (nAChRs) in the superficial dorsal horn of the spinal cord. Nonetheless, the functional significance of nAChRs in the deep dorsal horn of adult animals remains unclear. Using whole-cell patch-clamp recordings from lamina V neurons in the adult rat spinal cord, we investigated whether the activation of nAChRs could modulate the inhibitory synaptic transmission in the deep dorsal horn. In the presence of CNQX and APV to block excitatory glutamatergic synaptic transmission, bath applications of nicotine (100 μM) significantly increased the frequency of spontaneous inhibitory postsynaptic currents (sIPSCs) in almost all neurons tested. The effect of nicotine was mimicked by N-methyl-4-(3-pyridinyl)-3-butene-1-amine (RJR-2403, 100 μM), an α4β2-nAChR agonist, and was also mimicked by choline (10 mM), an α7-nAChR agonist. The effect of nicotine was completely blocked by the nAChR antagonist mecamylamine (5 μM). In the presence of tetrodotoxin (0.5 μM), nicotine (100 μM) significantly increased the miniature IPSC frequency. On the other hand, RJR-2403 (100 μM) or choline (10 mM) did not affect miniature IPSCs. The application of nicotine (100 μM) also evoked a large inward current in all lamina V neurons tested when cells were held at -60 mV. Similarly, RJR-2403 (100 μM) induced inward currents in the majority of lamina V neurons examined. On the other hand, choline (10 mM) did not elicit any detectable whole-cell currents. These results suggest that several nAChR subtypes are expressed on the presynaptic terminals, preterminals, and neuronal cell bodies within lamina V and that these nAChRs are involved in the modulation of inhibitory synaptic activity in the deep dorsal horn of the spinal cord.

## Background

Neuronal nAChRs are a larger family of ligand-gated ion channels widely expressed in both the central and the peripheral nervous system. At least 12 different subunits of nAChRs, including α2–α10, β2–β4, have been identified so far and these subunits form many different subtypes of nAChRs with pentameric structures consisting of homomers or heteromers [1]. Homomeric nAChRs are made up of α7, α8 or α9 subunits, while heteromeric nAChRs comprise various combinations of α2–α6 with β2–β4 subunits, α9 with α10 subunits [2,3]. These subtypes of nAChRs have different pharmacological and biophysical properties [1]. It has been shown that nAChRs are involved in a variety of physiological functions including learning, reinforcement, development, aging and nociception [4].

Although Davis et al. (1932) first reported that nicotine has analgesic effects [5], high dosages of nicotine were required to produce antinociception and its effect was relatively modest with a short duration [6-8]. Epibatidine, a potent nAChR agonist isolated from the skin of an Ecuadorian frog, was about 100-fold more potent than morphine in rodents [9-12]. Unfortunately, the dosage of epibatidine to produce antinociception was near that to cause seizure, death, and other side effects [12]. The intolerable toxic effects of epibatidine were due to its actions on a broad range of nAChR subtypes. Therefore, the key to the development of safe and effective nicotinic agonists as analgesics is to first understand which nAChR subtypes are involved in modulating nociceptive transmission.

The spinal dorsal horn is the first site in the central nervous system where somatosensory information is processed and integrated. Multiple subtypes of nAChRs are expressed in the spinal dorsal horn and these receptors have been indicated to modulate sensory inputs from the periphery. Genzen and McGehee (2003) have demonstrated that the activation of α7 nAChRs located at the central terminals of primary afferents enhances the glutamatergic excitatory transmission in the spinal dorsal horn [13]. Several subtypes of nAChRs have been shown to exert tonic or phasic control on the descending inhibitory serotonergic transmission [14]. Multiple subtypes of nAChRs are found to be expressed on both inhibitory and excitatory interneurons in the spinal dorsal horn [15]. The activation of presynaptic nAChRs facilitates GABAergic and glycinergic inhibitory synaptic transmission in the superficial dorsal horn [16-19]. Although the roles of presynaptic nAChRs were extensively studied in the superficial dorsal horn, it is unclear whether nAChRs also mediate sensory modulation in the deep dorsal horn of the spinal cord in adult animals. A variety of sensory inputs, including nociceptive and non-nociceptive inputs, are transmitted into deep dorsal horn [20]. Deep dorsal horn neurons, especially those in the lamina V region, can generate long-lasting afterdischarges in response to nociceptive inputs and this hyperactivity has important implications in pathological pain states [21]. Inhibitory modulation in this region is critical in preventing the central hyperactivity and hyperalgesia. The aim of this study was to evaluate the effects of nAChR activation on the inhibitory synaptic transmission in deep dorsal horn neurons.

## Results

### Effects of nicotine and nAChR agonists on spontaneous IPSCs in the lamina V neurons

**Figure 1:**
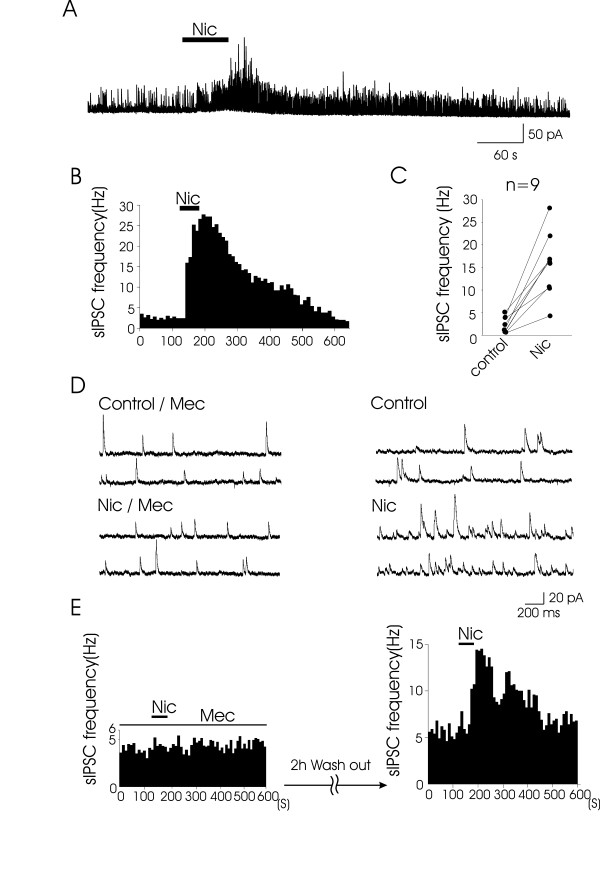
**Effects of nicotine on spontaneous IPSCs in lamina V neurons**. **A**, A continuous recording of sIPSCs in the control and following the application of nicotine (Nic, 100 μM). **B**, A histogram shows the time course of the changes in sIPSC frequency following the application of nicotine; time bin is 10 s. **C**, The graph shows the individual result from 9 lamina V neurons. **D**. Effects of mecamylamine (Mec) on nicotine-induced increase in sIPSC frequency. The consecutive traces on the left are sIPSCs in the control (upper panel) and following the application of nicotine (lower panel) in the presence of mecamylamine (5 μM). The consecutive traces on the right are sIPSCs in the control (upper panel) and following the application of nicotine (lower panel) after the washout of mecamylamine. Note that the bath application of nicotine did not affect the sIPSCs in the presence of mecamylamine, but it markedly increased sIPSC frequency after the washout of mecamylamine. **E**, Two histograms show time courses of changes in sIPSC frequency following the application of nicotine in the presence of mecamylamine (left) and after the washout of mecamylamine (right); time bin is 10 s.

Whole-cell patch-clamp recordings were performed from lamina V neurons of spinal cord slices prepared from adult rats. Stable recordings could be obtained from slices maintained *in vitro *for more than 12 hours. Glutamatergic excitatory postsynaptic transmission was blocked by CNQX (20 μM) and APV (50 μM). All lamina V neurons tested exhibited spontaneous inhibitory postsynaptic currents (sIPSCs) when cells were held at -10 mV. In the presence of bicuculline (20 μM) and strychnine (2 μM), sIPSCs were completely abolished in all lamina V neurons tested (*n *= 3; data not shown), indicating these sIPSCs were mediated by GABA and/or glycine receptors. Perfusion of nicotine (100 μM) for 1 min resulted in a rapid and significant increase in sIPSC frequency in all neurons tested (Fig. 1A–C). The average sIPSC frequency in controls was 2.1 ± 0.6 Hz (0.4 – 5.1 Hz, n = 9) and the frequency increased to 15.8 ± 2.3 Hz (4.2 – 28.1 Hz, n = 9, P < 0.05) following the application of 100 μM nicotine (Fig. 1C); the sIPSC frequency increased to 1330 ± 310% of the control (*n *= 9, P < 0.05). The nicotine-induced increase in sIPSC frequency was completely blocked in the presence of nAChR antagonist mecamylamine (5 μM) administrated 5 min prior to the application of nicotine (*n *= 3; Fig. 1D, E). After the washout of mecamylamine, a second application of nicotine (100 μM) increased the sIPSC frequency in all neurons tested (Fig. 1D, E).

**Figure 2:**
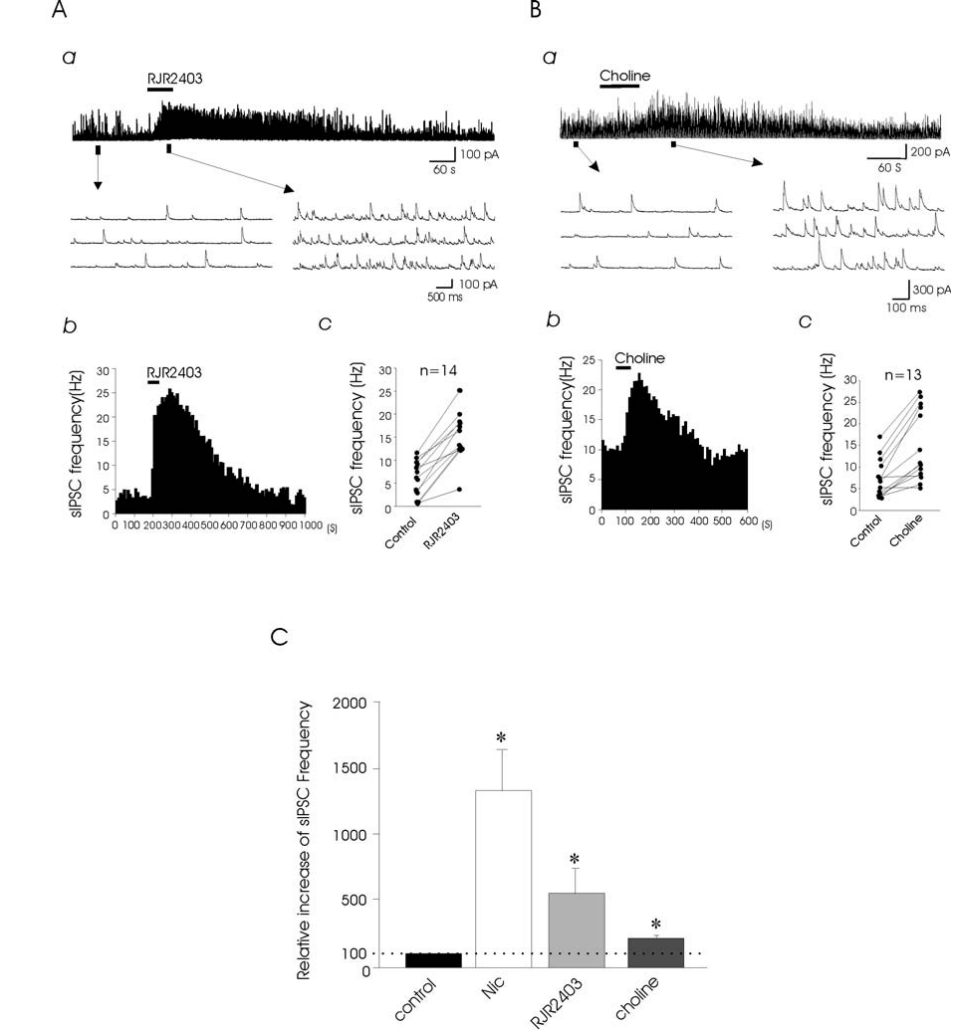
**Effects of nicotinic receptor agonists on sIPSCs in lamina V neurons**. **A**, **(a) **A continuous recording of sIPSCs in the control and following the application of the selective α4β2 nAChR agonist RJR-2403 (100 μM). **(b)**, A histogram shows the time course of changes in sIPSC frequency following the application of RJR-2403; time bin is 10 s. **(c)**, The graph shows the individual result from 14 lamina V neurons. **B**, The experiment was similar to that shown in **(A) **except that the selective α7 nAChR agonist choline (10 mM) was tested. Similar results were obtained in 11 out of 13 neurons. **C**, A histogram shows relative sIPSC frequency following the application of nicotine (*n *= 9), RJR-2403 (*n *= 14), or choline (*n *= 13). sIPSC frequency before the applications of testing drugs is used as control and is scaled at 100%. Data represent Mean ± SEM; *P < 0.05.

We tested RJR-2403, a selective α4β2 nAChR agonist, to see if it also increased sIPSC frequency. Similar to nicotine, application of 100 μM RJR-2403 for 1 min markedly increased sIPSC frequency in 13 out of 14 neurons recorded (Fig. 2Aa–c). The average sIPSC frequency in the control and following the application of RJR-2403 was 5.8 ± 1.0 Hz (0.5 – 11.6 Hz, n = 14) and 15.7 ± 1.9 Hz (3.7 – 25.2 Hz, n = 14), respectively (Fig. 2Ac). The sIPSC frequency following the applications of RJR-2403 significantly increased to 573 ± 189% of the control (*n *= 14, P < 0.05; Fig. 2C). While RJR-2403 alone produced a significant increase in sIPSC frequency, the effects of RJR-2403 was completely blocked in the presence of dihydro-beta-erythroidine (DhβE, 1 μM), an α4β2 nAChR antagonist (97 ± 2% of control, n = 3). Perfusion of choline (10 mM), a selective α7 nAChR agonist, for 1 min also increased the sIPSC frequency in 11 out of the 13 neurons examined (Fig. 2Ba–c). The average sIPSC frequency in the control and following the application of choline was 7.4 ± 1.2 Hz (2.9 – 16.9 Hz, n = 13) and 15.2 ± 2.3 Hz (5.2 – 27.6 Hz, n = 13), respectively (Fig. 2Bc). The sIPSC frequency following the applications of choline significantly increased to 221 ± 22% of control (*n *= 13, P < 0.05; Fig. 2C). While choline alone produced a significant increase in sIPSC frequency, choline did not produce any significant increase in sIPSC frequency in the presence of methyllycaconitine (MLA, 50 nM), an α7 nAChR antagonist (98 ± 2% of control, n = 4).

### Effects of nicotine and nAChR agonists on mIPSCs in lamina V neurons

**Figure 3:**
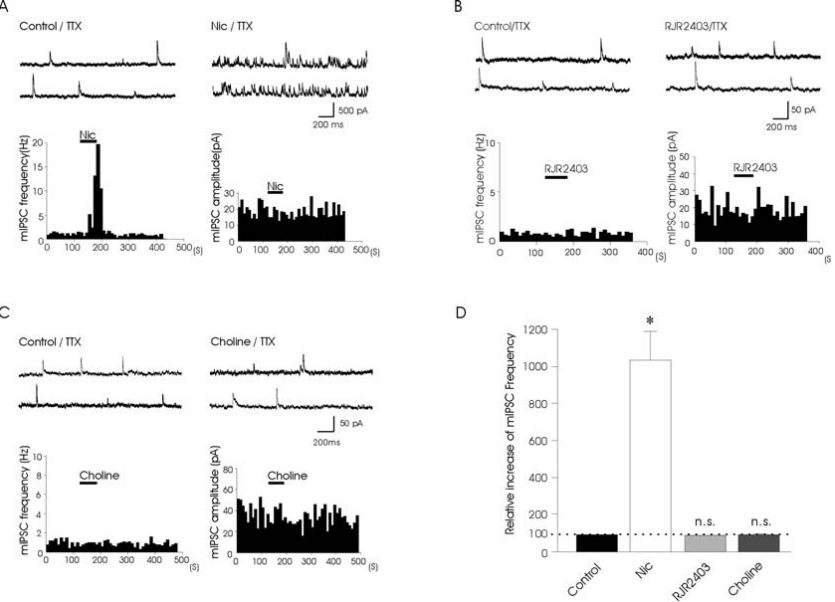
**Effects of nicotinic receptor agonists on mIPSCs in lamina V neurons**. **A**, The consecutive traces of mIPSCs are in the control (left) and following the application of nicotine (100 μM, right). Two histograms on the bottom panel show the time course of changes in mIPSC frequency (left) and amplitude (right) during the application of nicotine; time bin is 10 s. **B**, **C**, Experiments were similar to that shown in **(A) **except that 100 μM RJR-2403 or 10 mM choline was tested. **D**, A histogram shows relative mIPSC frequency following the applications of nicotine (*n *= 11), RJR-2403 (*n *= 6), or choline (*n *= 7). mIPSC frequency before the applications of testing drugs is used as control and is scaled at 100%. Data represent Mean ± SEM; *P < 0.05; n.s., not significant.

We examined the effects of nicotine or nAChR agonists on mIPSC frequency in the presence of tetrodotoxin (TTX, 0.5 μM) to determine whether nAChRs might be localized at the presynaptic terminals of GABAergic and/or glycinergic inhibitory interneurons. Application of 0.5 μM TTX itself blocked the action potential-driven synaptic transmission and decreased the amplitude of IPSCs from 149.9 ± 125.6 pA to 30.0 ± 18.4 pA (n = 4). Under this condition, bath application of nicotine (100 μM) largely increased mIPSC frequency in all neurons recorded, but there was no effect on mIPSC amplitude (*n *= 10; Fig. 3A). The average mIPSC frequency in the control and following the applications of nicotine was 1.7 ± 0.4 Hz (0.4 – 5.3 Hz, n = 10) and 15.0 ± 2.3 Hz (1.4 – 25.2 Hz, n = 10), respectively. The mIPSC frequency following the applications of nicotine significantly increased to 1043 ± 153% of control (n = 10, P < 0.05, Fig. 3D). On the other hand, perfusion of 100 μM RJR-2403 (n = 6) or 10 mM choline (n = 7) did not affect mIPSC frequency and amplitude (Fig. 3B, 3C). The average mIPSC frequency following the application of RJR-2403 and choline was 95 ± 3% of control (*n *= 6) and 98 ± 2% of control (*n *= 7), respectively (Fig. 3D).

### Whole-cell currents directly evoked by nicotine or nAChR agonists in lamina V neurons

**Figure 4:**
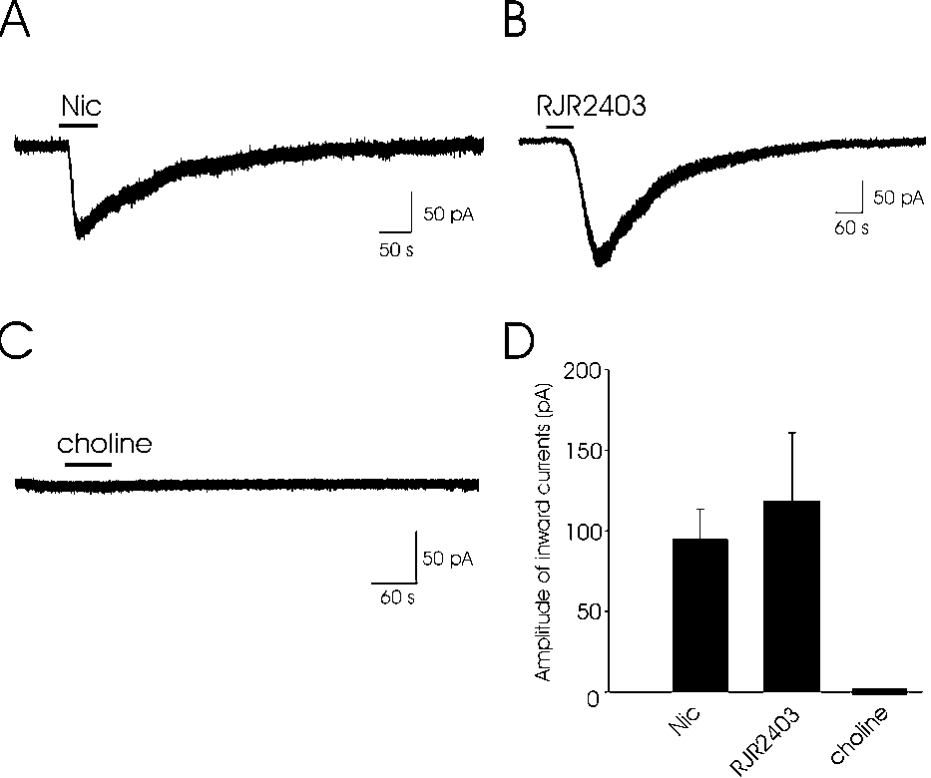
**Whole-cell currents evoked by nicotine and nAChR agonists in lamina V neurons**. **A**, Whole-cell currents evoked by bath application of nicotine (100 μM) in a lamina V neuron. **B**, Whole-cell currents evoked by RJR-2403 (100 μM) in a different lamina V neuron. **C**, Choline (10 mM) did not evoke any membrane current in a neuron. **C**, The histogram shows the average amplitude of the whole-cell inward currents induced by nicotine (*n *= 8), RJR-2403 (*n *= 6), and choline (*n *= 6).

We determined whether nicotine, RJR-2403 and choline could evoke whole-cell currents in lamina V neurons. In this set of experiments, cells were held at -60 mV and recordings were conducted in the presence of 20 μM CNQX, 50 μM APV, 20 μM bicuculline and 10 μM PMBA (3-[2'-Phosphonomethyl[1,1'-biphenyl]-3-yl]alanine). Under this condition, both excitatory and inhibitory postsynaptic currents were completely disappeared. The bath application of nicotine (100 μM) for 1 min evoked an inward current in all neurons tested (Fig. 4A). The average peak amplitude of the inward currents evoked by nicotine was 95 ± 19 pA (*n *= 8; Fig. 4D). The bath application of RJR-2403 (100 μM) for 1 min also evoked large inward currents in 6 out of 8 neurons examined (Fig. 4B). The average peak amplitude of the inward currents induced by RJR-2403 was 119 ± 42 pA (*n *= 6; Fig. 4D). In contrast to nicotine and RJR-2403, choline (10 mM) did not elicit any detectable currents (*n *= 6; Fig. 4C).

## Discussion

The present study demonstrated in lamina V neurons of the adult rat spinal cord that nicotine increased sIPSC frequency when glutamatergic excitatory transmission was blocked in the presence of CNQX and APV and that nicotine also increased mIPSC frequency when action potential-driven synaptic transmission was not permitted in the presence of TTX. Interestingly, however, neither the α4β2 nor α7 nAChR agonists increased mIPSC frequency although both of them increased sIPSC frequency in lamina V neurons. Together with the findings of our previous study conducted on superficial laminas of the spinal cord of adult rats [18], we have provided electrophysiological evidence showing that inhibitory synaptic activity in both superficial and deep laminas of the spinal cord dorsal horn are modulated by different nAChR subtypes.

**Figure 5:**
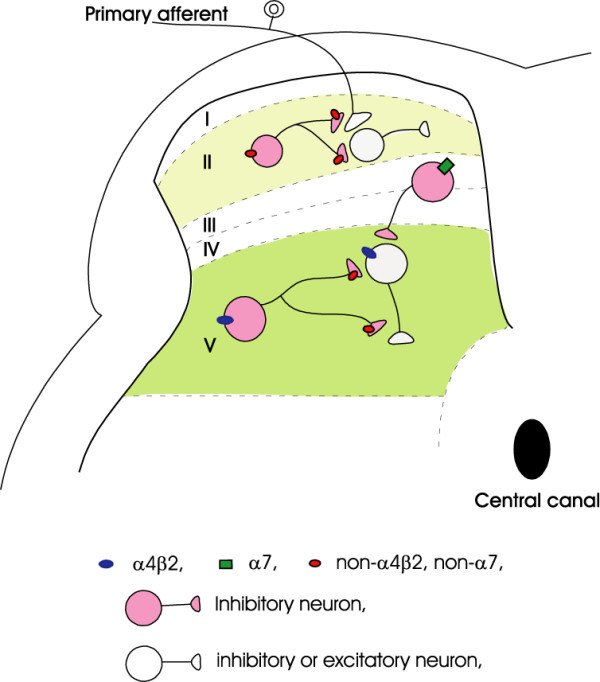
**Schematic diagram of nAChR-mediated modulcation of sensory synaptic transmission in the dorsal horn of adult rats**. In adult rats, α4β2 nAChRs are expressed on the soma of inhibitory interneurons located in the lamina V region. These nAChRs may be also expressed on preterminals but not at presynaptic sites of the lamina V inhibitory neurons. Lamina V neurons are also synapsed by inhibitory neurons expressing α7 nAChRs at their preterminals and/or on their somas and these inhibitory neurons are likely to be located in other lamina regions. The modulation of inhibitory activity in lamina V by both α4β2 nAChRs and α7 nAChRs depends on membrane depolarization and action potentials. There is a non-α4β2/non-α7 subtype of nAChRs that are expressed at the presynpatic terminals of inhibitory neurons in lamina V region. The modulation of inhibitory transmission in lamina V by non-α4β2/non-α7 subtype of nAChRs is independent of membrane depolarization and action potentials. The distribution of nAChR subtypes in the spinal cord lamina II region of adult rats [18] is also presented in this diagram for a comparison.

Nicotinic receptors are abundant in different CNS regions, where they are shown to regulate the release of various neurotransmitters, including serotonin, norepinephrine, glutamate, GABA and glycine [22-24]. In the present study, the activation of nAChRs enhanced the GABA and/or glycine release onto lamina V neurons. A similar enhancement of the inhibitory synaptic transmission by the activation of nAChRs has been reported in the superficial layers of the spinal dorsal horn [16,18,19]. In neonatal rats, α4β2 nAChR subtype has been suggested to be expressed at presynaptic terminals and these receptors mediate significantly increases in the glycinergic [16] and GABAergic inhibitory synaptic transmission in the superficial lamina of the spinal cord dorsal horn [19]. Interestingly, the expression of nAChR subunits in the spinal cord changes during development [25]. Consistent with the development changes of nAChR subunits, our previous study demonstrated that a non-α4β2 and non-α7 subtype nAChR mediated an enhancement of both the GABAergic and glycinergic mIPSC frequency in the superficial laminas of the adult rat spinal dorsal horn [18]. In the deeper laminas (lamina III–V) of neonatal rats, a previous study showed that presynaptic α4β2 nAChRs mediate the facilitation of GABA release [19], a result similar to those shown in superficial laminas of neonatal rats [16,19]. However, the present study revealed that the nAChR-mediated modulation of inhibitory synaptic transmission in the adult stage is more complicated than that in the neonatal stage. We showed that nicotine largely increased both sIPSC frequency and mIPSC frequency in the lamina V neurons in adult rats. On the other hand, RJR-2403, a potent activator of α4β2 nAChR, and choline, a selective agonist for α7 nAChR, significantly increased the sIPSC frequency, but did not change mIPSC frequency. It has been demonstrated that nAChRs are expressed at two cellular locations in the central nervous system [26]. One is presynaptic sites or synaptic boutons where nAChR activation modulates transmitter release in a TTX-insensitive manner. The other is preterminals at terminal axon branches where nAChR activation affects transmitter release by depolarizing axonal membranes to fire action potentials. A recent immunohistochemical study revealed the immunoreactivity of nAChRs in lamina V neurons of the spinal dorsal horn at both presynaptic and preterminal sites [27]. At the preterminal sites, nAChR-mediated regulation of transmitter releases is TTX-sensitive and can be blocked in the presence of TTX. The block by TTX of the RJR-2403- and choline-induced increase in sIPSC frequency in our results suggests that α4β2 and α7 nAChRs are not expressed at presynaptic terminals. These receptors are likely to be expressed at the preterminals or other parts of GABAergic and/or glycinergic neurons whose axons innervate lamina V neurons. Because nicotine could still increase IPSC frequency in the presence of TTX, it suggests that a non-α4β2, non-α7 subtype of nAChR is located at the presynaptic terminals of GABAergic and/or glycinergic neurons that innervate lamina V neurons (Fig. 5). The presence of a non-α4β2, non-α7 subtype of nAChR in spinal cord dorsal horns are supported by previous studies using α4β2 nAChR knock-out mouse [28], in situ hybridization [29] and the combination of patch-clamp recordings with single-cell RT-PCR [15]. All these previous studies pointed to the potential presence of other functional nAChR subtypes in addition to α4β2 and α7 receptors. However, the subunit compositions of the non-α4β2 and non-α7 subtype of nAChRs remain to be identified.

We have shown that both nicotine and the α4β2 nAChR agonist RJR-2403 directly evoked whole-cell inward currents in the majority of lamina V neurons. On the other hand, the α7 nAChR agonist choline did not evoke any detectable inward currents in lamina V neurons. Since strychnine has been noted to also be an effective antagonist at α7 nAChRs [30,31], the effect of choline was examined in the presence of PMBA, a glycine receptor antagonist that has no effect on α7 nAChRs [32,33]. The lack of choline-evoked whole-cell currents raise a possibility that α7 nAChRs are not expressed on lamina V neurons. If this is the case, then choline-induced increases of sIPSC frequency might be mediated by α7 nAChRs that are expressed on the inhibitory interneurons whose cell bodies are located in other lamina regions in the spinal cord. In contrast to α7 nAChRs, our results suggest that α4β2 nAChR expressing inhibitory interneurons are located in lamina V. Consistently, several reports have indicated the presence of α4β2 nAChR in the deep dorsal horn and α7 nAChRs in other lamina of the spinal cord [15,33-35]. Bradia et al. (2002) has reported that α-bungarotoxin-sensitive α7 nAChRs are located in the parasympathetic preganglionic neurons surrounding the central canal of the spinal cord (lamina X) [33]. A low level of α7 transcripts were also detected by *in situ *hybridization in the area around the central canal [35]. Moreover, the single-cell RT-PCR study revealed a more widespread expression of α7 nAChR subunits in mouse spinal dorsal horn neurons [15]. These findings support the idea that α7 nAChR-expressing inhibitory interneurons innervate lamina V neurons from other lamina regions in the spinal cord.

The role of nAChRs in modulating pain transmission has been reported by a number of studies. Using α4β2 knock-out mice, Marubio et al. (1999) showed a reduced antinociceptive effect in a behavior study [28]. In a neuropathic mouse model, epibatidine, a potent agonist of nAChRs showed strong analgesic effects. However, the effects of epibatidine were not completely prevented by the α4β2 nAChR antagonist dihydro-β-erythroidine [36]. These studies suggested that in addition to α4β2 nAChR, other nAChRs were involved in nAChR-mediated analgesic effects. Consistent with this idea, the intrathecal injection of choline, an α7 nAChR agonist, has been reported to have an antinociceptive effect [37]. Several mechanisms have been proposed to contribute to nAChR-mediated analgesic effects, including the desensitization of nAChRs on nociceptive primary afferent fibers, the increase of noradrenaline and serotonin release within the spinal cord, the activation of the descending inhibitory pathways [14,38], and the increases of GABA and glycine release from inhibitory interneurons in the superficial spinal cord dorsal horn [39]. Our study suggest that α4β2, α7 nAChRs, and another undefined subtype of nAchRs are involved in regulating GABA and/or glycine release in the deep lamina of the spinal cord dorsal horn in adult animals.

In this study, the application of nicotine or nicotinic agonists significantly facilitated GABAergic and/or glycinergic inhibitory synaptic transmission in the deep dorsal horn of the spinal cord. This raises a possibility that acetylcholine released endogenously may modulcate inhibitory synaptic transmission in a similar fashion. Recently, Rashid et al. (2006) suggested that endogenous acetylcholine tonically stimulated the GABA and glycine release via α4β2 subtype of nAChRs in the superficial dorsal horn in mice [40]. Acetylcholine may be released from the interneurons in the dorsal horn since the cell bodies of cholinergic interneurons have been found in lamina III–V [41]. It appears that in the deep dorsal horn there are no descending cholinergic systems in the rat [20,42]. Thus, cholinergic interneurons in the dorsal horn [43,44] may play an important role in modulating inhibitory synaptic transmission.

In the dorsal horn, GABAergic and glicinergic inhibitory synapses undergo developmental changes [45-47]. In the present study, we did not separate inhibitory activity between those of GABAergic synapses and those of glycinergic synapses. It would be interesting to further study whether nAchR subtype expression on GABAergic and glycinergic neurons is different in the spinal cord dorsal horn.

## Conclusion

We have demonstrated that several nAChR subtypes are expressed on the presynaptic terminals, preterminals, and neuronal cell bodies within lamina V and that they are involved in the facilitation of inhibitory synaptic transmission. Therefore, the activation of nAChRs in the deep dorsal horn of the spinal cord may be capable of inhibiting nociceptive signaling in physiological and pathological pain sensations.

## Methods

All the experimental procedures involving the use of animals were approved by the Ethics Committee on Animal Experiments, Wakayama Medical University, and were in accordance with the UK Animals (Scientific Procedures) Act 1986 and all associated guidelines.

### Spinal cord slice preparation

The method used to prepare adult rat spinal cord slices has been described previously [48]. In brief, male adult Sprague-Dawley rats (6–8 weeks of age, 200–300 g) were deeply anaesthetized with isoflurane through a nose cone inhalation, and then lumbosacral laminectomy was performed. The lumbosacral spinal cord (L1-S3) was removed and placed in pre-oxygenated Krebs solution at 1–3°C. Immediately after the removal of the spinal cord, the rats were killed by exsanguination. The pia-arachnoid membrane was removed after cutting all the ventral and dorsal roots near the root entry zone. The spinal cord was mounted on a vibratome and then a 600 μm-thick transverse slice was cut. The slice was placed on nylon mesh in the recording chamber, which had a volume of 0.5 ml, and then was perfused at a rate of 10–15 ml/min with Krebs solution saturated with 95% O_2 _and 5% CO_2_, and maintained at room temperature. A platinum grid was placed on the top of the slice to prevent slice movement. The Krebs solution contained (in mM) 117 NaCl, 3.6 KCl, 2.5 CaCl_2_, 1.2 MgCl_2_, 1.2 NaH_2_PO_4_, 25 NaHCO_3 _and 11 glucose.

### Patch-clamp recordings from lamina V neurons

Blind whole-cell patch-clamp recordings were made from lamina V neurons with patch-pipette electrodes having a resistance of 5–10 MΩ [48]. The patch-pipette solution was composed of (in mM) 110 Cs_2_SO_4_, 5 Tetraethylammonium (TEA), 0.5 CaCl_2_, 2 MgCl_2_, 5 EGTA, 5 HEPES, 5 ATP-Mg, pH 7.2. Signals were acquired with a patch-clamp amplifier (Axopatch 200B; Axon Instruments, Foster City, CA, USA). The data were digitized with an A/D converter (Digidata 1200, Axon Instruments) and stored and analyzed with a personal computer using the pCLAMP data acquisition program (Version 8.2, Axon Instruments). Lamina V neurons were viable for up to 24 h in slices perfused with a pre-oxygenated Krebs solution. All the recordings described in this study were made within 12 h. Whole-cell patch-clamp recordings were stable for up to 4 h. All of the neurons had membrane potentials more negative than -50 mV. Unless otherwise noted, all the recordings in this study were performed in the presence of CNQX (20 μM) and APV (50 μM).

### Drug Applications

Drugs were dissolved in Krebs solution and then were applied by perfusion via a three-way stopcock without any change in the perfusion rate or the temperature. The time necessary for the solution to flow from the stopcock to the surface of the spinal cord slice was approximately 20 s. The drugs used in this study were nicotine (Sigma-Aldrich, St. Louis, MO, USA), RJR2403 (Tocris, Ballowin, MO, USA), choline (Sigma-Aldrich), mecamylamine (Sigma-Aldrich), 3-[2'-Phosphonomethyl[1,1'-biphenyl]-3-yl]alanine (PMBA, Sigma RBI), bicuculline (Sigma-Aldrich), strychnine (Sigma-Aldrich), 6-cyano-7-nitroquinoxaline-2,3-dion (CNQX, Tocris), D(-)-2-Amino-5-phosphonopentanoic acid (D-APV, Tocris), and tetrodotoxin (TTX, Tocris).

### Statistical analysis

All numerical data were expressed as the mean ± S.E.M. Statistical significance was determined as P < 0.05 using paired Student's t-test. For electrophysiological data, *n *refers to the number of neurons recorded.

## Abbreviations

nACh, nicotinic acetylcholine receptor;

IPSC, inhibitory postsynaptic current;

RJR2-403, N-methyl-4-(3-pyridinyl)-3-butene-1-amine;

TTX, tetrodotoxin;

GABA, gamma-aminobutyric acid;

TEA, tetraethylammonium;

EGTA, ethyleneglycol bis(2-aminoethylether)tetraacetic acid;

HEPES, N-(2-Hydroxyethyl)piperazine-N'-(2-ethanesulfonic acid);

ATP-Mg, adenosine triphosphate-magnesium;

CNQX,6-cyano-7-nitroquinoxaline-2,3-dion;

PMBA,3-[2'-Phosphonomethyl[1,1'-biphenyl]-3-yl]alanine;

D-APV, D(-)-2-Amino-5-phosphonopentanoic acid;

## Competing interests

The author(s) declare that they have no competing interests.
